# Implementing service transformation for children and adolescents with eating disorders across England: the theory, politics, and pragmatics of large-scale service reform

**DOI:** 10.1186/s40337-022-00665-z

**Published:** 2022-10-10

**Authors:** Ivan Eisler, Mima Simic, Peter Fonagy, Rachel Bryant-Waugh

**Affiliations:** 1 Maudsley Centre for Child and Adolescent Eating Disorders (MCCAED), Maudsley Hospital, De Crespigny Park, Denmark Hill, London, SE5 8AZ UK; 2 Institute of Psychiatry, Psychology and Neuroscience, King’s College London, London, UK; 3 Research Department of Clinical, Educational and Health Psychology, University College London, London, UK; 4 Anna Freud National Centre for Children and Families, London, UK

**Keywords:** Child, Adolescent, Eating disorders, Evidence based practice, Service transformation, Care pathway, Expert community-based treatment, Multi-disciplinary team

## Abstract

**Background:**

Eating disorders are among the most serious mental health problems affecting children and young people and without appropriate treatment often have a protracted course with high levels of morbidity and mortality. While considerable progress has been made in recent years in developing effective evidence-based outpatient treatments, these are not always readily available. In England, until recently, the usual care pathway for young people with an eating disorder was referral from primary care to local generic Child and Adolescent Mental Health Services with varying levels of expertise in eating disorders and a mix of outpatient treatments available. Poor treatment progress or physical deterioration would usually result in inpatient admission. Admission rates were high, with children and young people with an eating disorder accounting for nearly a quarter of all child and adolescent psychiatric hospital admissions. Inpatient treatment is costly and has high relapse rates with some evidence that it may contribute to poorer long-term outcomes in eating disorders. Accumulating clinical and research evidence that early expert outpatient treatment can significantly reduce the need for inpatient care indicates,+ that investing in dedicated community-based eating disorders services is likely to be both clinically and economically beneficial.

**Overview of paper:**

This paper describes a large-scale transformation programme following a major government investment (initially £30 million/year, since then increased to over £50 million/year) aimed at service level change in the provision of eating disorder services for children and adolescents in England. We describe the history, background, political context, and clinical and research evidence that contributed to the government's decision to invest in eating disorders. We also provide a brief account of the implementation of an England-wide whole team training to support the creation of a network of over 70 dedicated community-based eating disorders services for children and young people.

**Supplementary Information:**

The online version contains supplementary material available at 10.1186/s40337-022-00665-z.

## Plain English summary

Eating disorders are among the most serious mental health problems affecting children and young people. There has been accumulating clinical and research evidence that early expert outpatient treatment is effective and can also significantly reduce the need for costly inpatient care, indicating that investing in community-based eating disorder services is likely to be both clinically and economically beneficial. This paper describes a large-scale service transformation programme following a major government investment (initially £30 million/year, since then increased to over £50 million/year) aimed at service level change in the provision of eating disorder services for children and adolescents in England. We describe the history, background, political context, and clinical and research evidence that contributed to the government's decision to invest in new services for eating disorders. We also provide a brief account of the implementation of an England-wide whole team training to support the creation of a network of over 70 dedicated community eating disorders services for children and young people.

**Supplementary Information:**

The online version contains supplementary material available at 10.1186/s40337-022-00665-z.

## Background

This paper describes a large-scale, England-wide, service transformation programme following a major government investment aimed at service level change in the provision of eating disorder (ED) services for children and adolescents in England [1]. We describe the history, background, political context, and clinical and research evidence that contributed to the government's decision to invest in EDs. We also provide a brief account of the implementation of an England-wide whole team training, supported by a separate government grant.

EDs are a major health problem, with high levels of morbidity and significant mortality [2, 3]. EDs have huge impacts not only on the sufferer and their families but also have high costs for society as a whole. In England, it has been estimated that direct health service costs for anorexia nervosa are between £40–230 million/year [4] with wider societal costs considerably higher with one study estimating them as £1.3–9.6 billion [5]. Similar or higher health economic and societal costs of eating disorders have been reported in recent studies from a number of other countries [6–8]. Direct health service costs of course vary considerably depending on the specific contexts of individual health care systems but evidence from different countries confirms that high health service costs are largely accounted for by hospital admissions [9–11]. In England, it has been estimated that inpatient costs may account for 75% of the total service costs [4].

Efforts to mitigate the impact of EDs have focused primarily on the development, evaluation, and dissemination of manualised, evidence-based treatments. The supporting evidence has been steadily growing over the past 30–40 years and while the quality of this evidence has been questioned [12, 13] there is a fair degree of agreement in the field as to which treatments are likely “frontrunners” [14]. For example, there is broad consensus, reflected in systematic reviews and clinical guidelines [15–18] that for children and adolescents, outpatient family therapy with a specific eating disorder focus has the strongest supporting evidence and should be recommended as the first-line treatment for both anorexia nervosa and bulimia nervosa.

Notwithstanding this consensus, there is a significant lag in the dissemination of clinical guidelines and practice in healthcare both generally [19] and specifically in EDs [20]. This is variously attributed to general scepticism among many clinicians about the use of manualised treatments [21], difficulties in providing effective training in evidence-based treatments for sufficient numbers of clinicians [22] and/or poor adherence to treatment manuals [23, 24].

The assumption that the key to better outcomes depends primarily on improving the transfer of the (fairly narrowly defined) research findings to clinical practice, however, has its limitations. While it is important, it is by no means the whole story as we will illustrate below.

### Common or non-specific factors in psychotherapy

One of the frequently voiced criticisms of the notion of *evidence-based treatments* (as opposed to the broader notion of *evidence-based practice—*[25, 26] is that specific treatment factors account for only a small proportion of outcome variance, according to some authors as low as 15% [27] or even less [28]. This is supported by research findings that adherence to manualised treatments has a limited impact on improving treatment outcomes [29, 30]. A recent systematic review and meta-analysis of psychological treatments for children and adolescents found that adherence to manuals was significantly correlated with outcome but accounted for only one percent of outcome variance [30].

There has been a long-standing debate in the psychotherapy literature about the relative contribution of specific and non-specific treatment factors [28, 31–33] although relatively few authors have addressed this in the EDs literature [34–36]. These debates have often been quite polarised which can be unhelpful [37]. Many would argue that both specific and non-specific treatment factors contribute to therapy outcomes [38] and that the two do not exist in isolation but interact with each other in ways that may improve or diminish clinical outcomes [39]. However, if we accept that non-specific treatment factors may have a significant role in the process of change, then any discussion of how to reduce the burden of EDs needs to go beyond considerations of how best to disseminate recommendations and supporting practice based solely on the results of randomised control trials (RCTs). Our aim is to highlight that how services are organised can act as a powerful non-specific treatment factor that can enable and amplify the process of therapeutic change targeted by *evidence-based treatments*. This has important implications for how effective, *evidence-based practice* is best disseminated.

### UK health service context of treatment of eating disorders

The UK has a National Health Service (NHS) free to users at the point of delivery and funded through taxation. The management of the NHS is devolved to the four national governments with England, Northern Ireland, Scotland and Wales each having their own health care systems. This means that in England (the focus of this paper) most of the treatment for EDs is provided through NHS England funding. Private health care providers are also available, but these are accessed by relatively few individuals, although private inpatient facilities for EDs receive significant funding from the NHS under contracts used when there is insufficient bed capacity within the public health system. Since its inception in 1948, the NHS funding mechanisms have frequently changed but since 2012 most of the decisions for commissioning mental health services in England have been devolved to 200 + Clinical Commissioning Groups (CCGs) (each covering on average a population of around 225,000).

Historically, the main treatment care pathway for young people with EDs available in England was by referral from primary care to a local Child and Adolescent Mental Health Service (CAMHS) that would have varying levels of expertise in EDs and a variable mix of outpatient treatments available. Poor progress in treatment or physical deterioration would indicate the need for more specialist or more intensive treatment which in most cases would be provided through inpatient admission. Within this context, rates of admission were high, 40–50% [40, 41].

The high level of hospital admissions for EDs meant that these represented nearly a quarter of all adolescent psychiatric hospital admissions [42] and because admissions for EDs were usually significantly longer than for other mental health problems, they accounted for approximately a third of bed occupancy [42–44]. The high rates of hospital admissions were difficult to justify on clinical grounds, given the compelling evidence that most children and adolescents with an eating disorder can be treated effectively on an outpatient basis [16]. Moreover, inpatient treatment for EDs, particularly where it is not supported by effective evidence-based post hospital treatment, while effective in the short term, has relapse rates of 25–30% after the first admission and 60–75% for second or further admissions [45, 46]. In comparison, effective outpatient treatment has shown in follow-up studies of RCTs to have relapse rates between 5 and 10% [47–49].

### Maudsley Centre for Child and Adolescent Eating Disorders as an exemplar of an alternative care pathway

The Maudsley Centre for Child and Adolescent Eating Disorders (MCCAED) evolved from a small clinical research team at the Institute of Psychiatry, Maudsley Hospital in London in the 1980s [50]. In 1995 the first dedicated community-based Child and Adolescent Eating Disorders Service was set up at the Maudsley Hospital. Initially, this was a small team providing outpatient treatment which was informed by findings showing the efficacy of family therapy for EDs. Over 90% of referrals were treated purely as outpatients with audit data showing good clinical outcomes and relatively low relapse rates (a recent case series and 7-year follow-up shows results that compare favourably with findings reported in RCTs [51, 52]). As the service expanded into a more comprehensive multidisciplinary team (including psychiatry, psychology, family therapy, paediatrics, nursing, dietetics) it established itself as the main treatment provider for child and adolescent EDs for seven London boroughs covering a population catchment area of approximately 2.2 million. By the early 2000s, a small number of similar services had appeared elsewhere in England, with anecdotal reports suggesting that setting up a dedicated outpatient service could reduce the number of hospital admissions by 80–90% [53]. These anecdotal experiences prompted the setting up of two important studies, the TOuCAN trial [54] and the London Care Pathways study [40], to evaluate the potential benefits of alternative, more specialist care pathways serving local populations.

### TOuCAN trial and London Care Pathways study

Gowers and colleagues [54], using a three-arm randomised design, compared outpatient treatment by non-specialist teams, outpatient treatment by specialist EDs teams, and inpatient care for the treatment of adolescent anorexia nervosa (age 12–18). The main clinical findings were that inpatient care was no more effective than outpatient treatment, but also no differences were found between the clinical outcomes of outpatient treatment delivered by specialist or non-specialist teams. A different picture emerged when health economic outcomes were considered, showing that treatment by specialist outpatient teams had the highest probability of being cost-effective [9]. Levels of satisfaction with treatment were also highest in those randomised to specialist EDs outpatient teams [55]. The conclusions of the study were limited by relatively poor adherence to the randomisation groups, particularly for those randomised to inpatient care (51% rejecting admission) and the fact that the main treatment in the specialist arm was a relatively brief CBT based programme rather than the now recommended anorexia nervosa focussed family therapy.

**Fig. 1:**
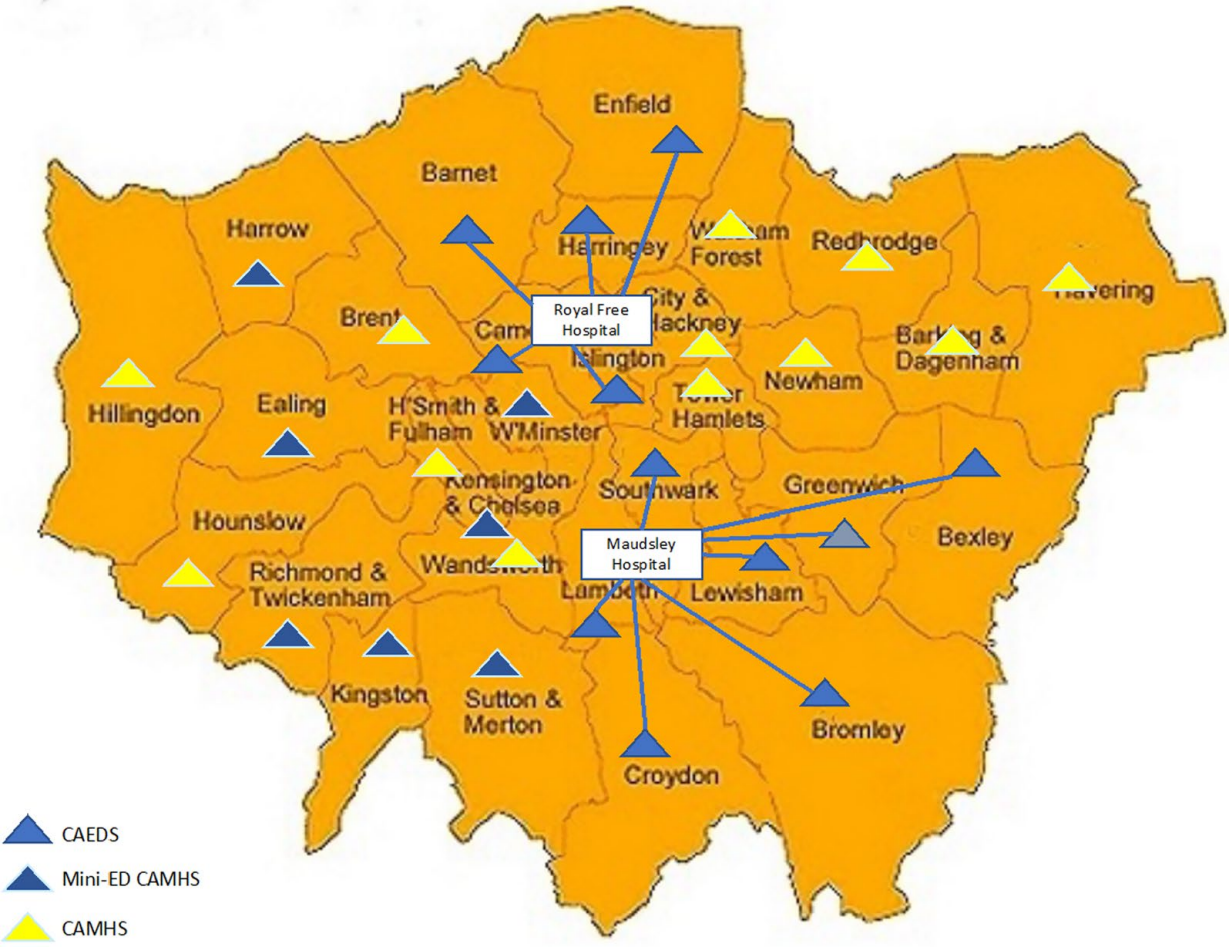
Child and adolescent EDs services in London boroughs in 2010. CAEDS—Dedicated community-based Child and Adolescent Eating Disorders Service. Mini-ED CAMHS—Mini eating disorder teams within generic CAMHS. CAMHS—Generic Child and Adolescent Mental Health Service

The London Care Pathway study [40] used a naturalistic design to evaluate the impact of different care pathways for adolescents with an EDs (aged 13–18). It made use of the fact that in some London boroughs there was direct access to dedicated community based EDs services from primary care whereas other areas used the more common non-specialist care pathway where initial referral from primary care was to a generic CAMHS service (Fig. 1).

The results showed striking differences in:

- Case identification 2.5 times higher in areas served by specialist teams.
- Reduction in hospitalisations by more than 50% in areas with specialist teams.
- Considerably higher consistency of care in specialist services where over 80% were assessed and treated within the same service compared to less than 20% of those initially referred to CAMHS.

**Fig. 2:**
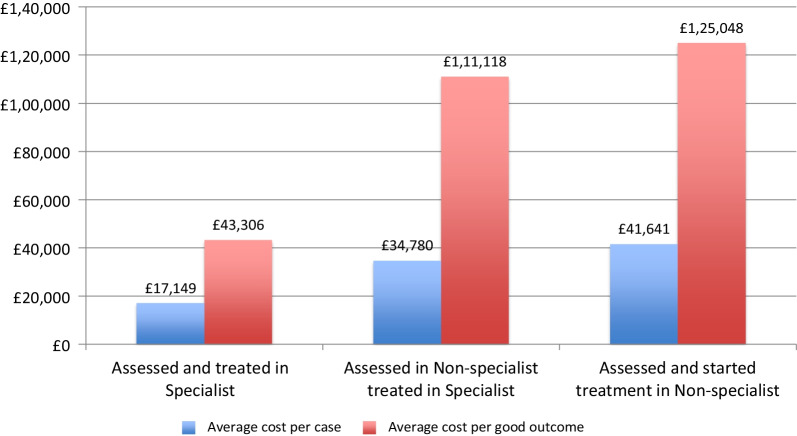
Direct healthcare cost by care pathway

**Fig. 3:**
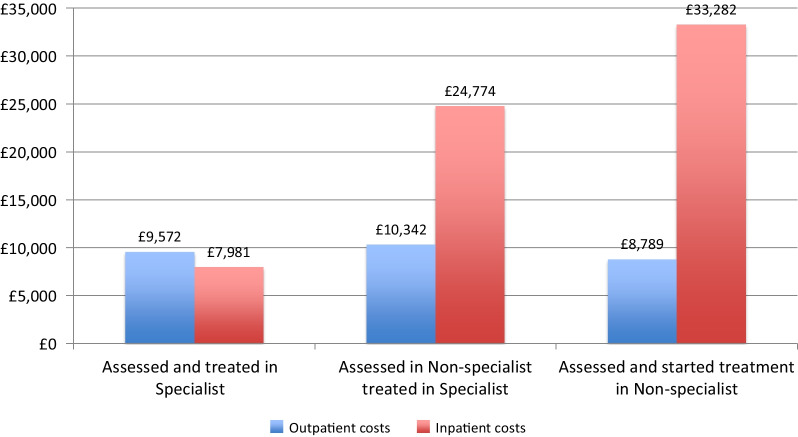
Average outpatient and inpatient costs by care pathway

A health economic analysis of the London Care Pathways study [4, 40] estimated that the average one-year health service costs for those who started treatment in specialist out-patient care to be approximately £17K rising to nearly £35K, for those assessed in CAMHS and immediately referred to specialist care and over £41K for those assessed and initially treated in generic CAMHS (Fig. 2). When the proportion of cases reaching a good outcome at one year is considered, the cost differences were even greater. The difference in treatment costs is nearly entirely accounted for by the varying levels of inpatient care (Fig. 3).

### Political and broader health service context

In November 2010 Commissioning Support for London (an NHS service set up to support London health service commissioners) invited MCCAED staff to present the (at that time unpublished) data from the London Care pathway study to London commissioners. In the discussion there was strong support from commissioners for the need for change and MCCAED and other community-based specialist services were seen as offering a template for developing other services. Furthermore, MCCAED and Royal Free Hospital CAEDS had recently set up intensive day programmes to complement their outpatient work and London was also well provided with specialist inpatient EDs care for the small numbers who would still require hospital-based treatment. London was therefore ideally placed to be used as a pilot for providing a new model of service provision for EDs in children and adolescents. In March 2011 a consultation document recommended changes in commissioning EDs services in London to include the following:

- Support the development of further three specialist community-based CAED services (South West London; Central and North West London; East London) each covering a population between 1–2 million.
- Develop a strong liaison between CAEDS and paediatric services to allow joint medical and EDs care for the most severely ill.
- Ensure close collaboration between CAED services and inpatient units when admissions are required, with CAED services closely involved in setting clear treatment goals and regular reviews to avoid unnecessarily long admissions.

Despite strong support from health service commissioners for the proposals, the broader political context meant that the proposal was shelved, and it took another four years before any change was initiated, this time aimed at the whole of England rather than the more modest proposal for piloting the changes just in London. In hindsight three main factors held back the process in 2011:

#### (a) Change in government and wider political context

In May 2010 a new government had been elected with a different vision of the NHS from the previous government. There was a new Secretary of State for Health, who embarked on a major reform of the NHS at the very time that the proposal for EDs services was put forward.

#### (b) Lack of published service level evidence supporting the need for change

At the time of the proposal, neither the cost analysis data from the London Care Pathway study nor audit data from MCCAED or other services in London had been published and the lack of such data considerably weakened the proposal. In hindsight, it would have been better to wait until such data were available.

#### (c) Lack of a clear voice from carers and families advocating for change in services

EDs were not high on the political agenda at the time and while many parents/carers reported dissatisfaction and difficulty accessing appropriate treatment, insufficient work had been conducted to capture the power of their collective voices. Occasional stories in the media typically concerned the most severely ill individuals which reinforced public perception that “real” treatment for EDs required hospitalisation and that investment was therefore needed to increase the number of beds.

Politically driven health service reforms often have unintended consequences, the impact of which may take considerable time to fully play out. The central aspect of the 2012 NHS reform was to transform the process of commissioning services by setting up a new commissioning body (NHS England) which was to be independent of but accountable to the Department of Health. The aim was to provide a clear structure in which NHS England was to have overall responsibility but devolved most of the service commissioning decisions to the local CCGs. Crucially in relation to this overview, the funding of the most specialised services (primarily inpatient services), was to be managed centrally by NHS England to ensure high level, uniform standards across the country. While this had some clear advantages, the disconnect between clinical decisions made locally and funding responsibility held centrally created, in many instances, unintended incentives for local services to admit to hospital, particularly as national austerity policies, following the 2008 financial crash, were leading to service restrictions in the NHS including CAMHS. The increase in the use of hospitalisation inevitably resulted in shortages of beds and growing waiting lists. Cuts in funding also meant that local CAMHS were less responsive and, particularly in the case of EDs, more likely to refer for inpatient treatment. Frequent press reports started to appear of patients with anorexia nervosa being told that they are not ill enough to receive treatment and/or patients being admitted to hospital at long distances from home. There was growing criticism of general underfunding for mental health and particularly for children and adolescents, with EDs being among the most visible and most often quoted examples.

## Investment in new service treatment provision

In 2014 two important government reports were published, one mapping CAMHS inpatient service provision [56] and the other reviewing Child and Adolescent Mental Health Services [57]. In June 2014 MCCAED submitted to the House of Commons Health Committee (HCHC) a reworked commissioning proposal, now including the London Care Pathways study cost data and findings from a 7-year MCCAED service audit report. Both the HCHC and NHS England reports made recommendations that included the need to consider funding an easily accessible, specialist community-based care pathway for children and young people with an ED. In response to this, in December 2014 the government announced new funding of £30 million/year for the treatment of child and adolescent EDs.^1^

Following the announcement, an Expert Reference Group of ED professionals, commissioners, service users, and carers was set up by NHS England in March 2015 which refined recommendations for how the new funding should be used. The resulting guideline Access and Waiting Time Standard for Children and Young People with an Eating Disorder [1] recommended that CCGs commission dedicated Community Eating Disorders Services for Children and Young People (CEDS-CYP) that would have the capacity and skill-mix to deliver NICE-concordant treatments and care via trained, appropriately supervised and well-resourced multi-disciplinary teams. The guideline recommended that the CEDS-CYP should meet the following requirements:

- Receive a minimum of 50 new ED referrals a year.
- Cover a minimum general population of 500,000.
- Provide interventions to treat both the ED and the most common coexisting mental health problems (e.g. depression and anxiety disorders).
- Enable direct access to these services through self-referral and from primary care services, by-passing generic CAMHS.
- Include medical and non-medical staff with significant EDs experience.

A key principle was that these services should be easily accessible by direct referral from primary care or by self-referral and that treatment should be provided as early as possible (within one week for urgent referrals and four weeks for more routine referrals).

Funding for the new services was allocated from April 2016, creating a network of over 70 CEDS-CYP across England. Funding (allocated according to the size of the population covered by each service) allowed for both expansion/improvement to existing services where these existed, as well as the development of new services in underserved populations.

## Workforce development and training

An important part of the remit of the Expert Reference Group was to develop a training curriculum [58] to support the upskilling of the CEDS-CYP staff. As described earlier, there is consensus as to what are the most efficacious treatments for anorexia nervosa and bulimia nervosa in children and adolescents which might suggest that training in NICE compliant treatments should be the primary aim. There were several reasons why it was deemed necessary to adopt a broader approach to the training that focussed on whole team development rather than simply training individual clinicians in specific evidence-based treatments.

First, was the recognition that the implementation of evidence-based practice requires not just changes in the knowledge and skills of individual clinicians but also necessitates systemic changes that facilitate and provide ongoing support for the effective delivery of the treatments in the specific service setting. This includes “buy-in” from senior staff (both clinical and managerial), the setting up of appropriate supervision structures, and the fostering of a service culture of evidence-informed practice in the broadest sense which includes ongoing learning, staying abreast of evolving research evidence, and routine monitoring of outcome and feedback data [59–62].

Second, the treatment of EDs requires a broad understanding of both the physical and psychological aspects of EDs and a service context that can safely manage the risks associated with the illness. Moreover, EDs are associated with high levels of comorbidity which are not always addressed by ED specific treatments and additional treatment provisions are likely to be needed to address these.

Third, the findings from the London Care Pathways study [40] indicated that service level factors are likely to have a key role in determining the *treatment reach* (i.e. how many people get treatment and how early they get it) with a major impact on health economic outcomes, particularly if they can avert admissions to hospital and/or significantly reduce the length of inpatient treatments. Many possible factors could explain the differences between the specialist and non-specialist care pathways (earlier intervention, confidence of the service in working safely with seriously ill children, the credibility of the service in the eyes of the family, containment of anxiety in the system, etc.) but it is likely, that the use of well researched evidence-based treatments was only one of many factors contributing to the outcomes. This connects with the earlier discussion of the contribution that common or non-specific psychotherapy factors are said to have in treatment outcomes.

**Fig. 4:**
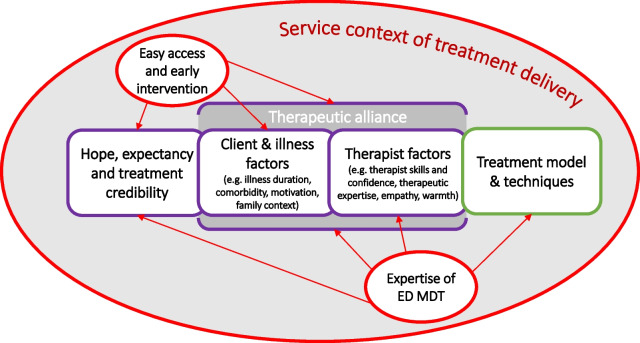
Putative impacts of a specialist service context on specific and non-specific therapy factors

A principle aim of the service transformation investment was to support the development of dedicated community-based EDs teams that would have the requisite knowledge and skills to deliver evidence-based interventions but at the same time also provide the context that would have the potential to shape and positively influence both the specific and the non-specific factors that are known to contribute to outcomes as illustrated in Fig. 4.

There were two central ideas that informed the nature of the service reorganisation. The first was that anyone with an ED at any level of severity (or just a suspected ED) should have direct access to a dedicated expert EDs service (which in the London Care Pathway study was associated with more than double the number of referrals compared to the non-specialist care pathway). The second was that the services had to be adequately resourced to be able to respond rapidly and offer treatment without delay. This is a service model common in physical health (e.g. cancer) but relatively rare in mental health. Such an approach is justified if early expert intervention reduces the likelihood of a more severe course of illness and avoids or reduces the need for other more costly treatments such as inpatient care. Both the high levels of expertise and early intervention are likely to impact a range of the non-specific therapy factors such as therapeutic alliance, hope and expectation, treatment credibility, motivation etc. (see Fig. 4). The multi-disciplinary team context provides support for individual therapists, contains anxiety and provides a safe context to deliver outpatient treatment even for more severely ill patients who would otherwise require treatment in an inpatient setting. The delivery of specific, evidence-based treatments is also enhanced by the context of a team that has a shared treatment philosophy reinforced by regular supervision and team case discussion.

## Key principles of the training programme

The key aim of the training was to develop *strong multidisciplinary teams with a broad knowledge of EDs and a shared, family-oriented, treatment philosophy* that would provide a base for disseminating and maintaining a high level of skill in delivering specific evidence-based treatments recommended by NICE guidelines. In addition to the points discussed above, a focus on training whole teams is likely to maximize learning, allowing for the best use of adult learning principles, where individuals share their learning and support each other in owning their existing expertise. It was also aimed at helping to evolve team cultures towards evidence-based practice as a broad overarching principle rather than a narrow focus on the delivery of a specific treatment. An important part of the basic team training, therefore, was to explore with teams their ongoing team development, further training and supervision needs and how these would be met both within the team and with additional focussed training in specific treatments.

Many of the skills needed to deliver comprehensive evidence-based treatments for EDs are generic skills adapted to the needs of the specific client group. The levels of these skills (e.g. individual or family therapeutic skills) and the knowledge base needed to deliver the treatment (e.g. knowledge about EDs, nutrition, and safe management of severally ill young people) will vary between different members of a team and an important part of the team development was to address how the existing expertise of different team members can be best utilised and brought together with the additional specialist expertise provided by the training.

The curriculum reflected the fact that the strongest evidence for effective treatments for young people with an ED is for ED focused family interventions, a central feature of which is the active engagement of families in helping to manage the eating of the ill person, while ensuring that the specific concerns and psychological needs of the young person are also met. The curriculum also recognised that while family interventions may have a central role in the treatment, other therapies such as CBT would be required, in some cases as an alternative for some patients and equally importantly as additional treatments to manage comorbid problems such as anxiety or depression or self-harming behaviours.

### The aims of the training

1. To implement the general principles of mental health services as part of the broader programme of Children and Young People’s Improving Access to Psychological Therapies (CYP IAPT) in the NHS [63]:
  - Full partnership and collaboration with children, young people and parents and carers in all aspects of care and service delivery.
  - Regular use of audit, outcome and feedback measurement to guide treatment and delivery and service development.
  - Improve early access to evidence-based treatments and services.
2. To ensure that by the end of the training the participants would have the essential knowledge about the nature of EDs including its physical and psychological effects, the course of the illness and its impact on young people and their families and to have the skills within the team for safe and effective management of the ED.
3. It was expected that knowledge would be held within the team as a whole but not necessarily in a detailed way by each team member and the training focus was therefore on the whole team of clinicians delivering psychological treatments, dietetic and paediatric staff, and others directly involved in direct care as well as administrative staff, managers and healthcare commissioners
4. An understanding and safe management of transitions between services (e.g. from community-based treatment to inpatient care and back to the community; care support in school; transition to adult services) as well as discharge back to primary care.
5. Knowledge of the range of psychological interventions for EDs and their evidence base. It was recognized that knowledge of therapies is not equivalent to being able to offer these treatments and the whole team training was aimed at identifying for each team the additional training needs if they are to deliver the full complement of NICE concordant ED treatments.
6. The embedding of supervision, consultation, and training structures within the team to ensure ongoing learning and skills development informed by the evolving evidence base for best practice.

### The structure and organization of the training

The challenge for the training was not just the numbers to be trained (more than 900 staff in 72 services^2^ across England) but also the considerable variability in the teams in terms of size, multidisciplinary composition, and existing expertise and knowledge of EDs. At one end there were small, newly developing teams setting up from scratch with many of the staff having quite limited EDs experience, and at the other end, well-established teams using the new funding to grow their team further but able to build on often many years of experience as a dedicated EDs service.

The variability in team composition, knowledge, and skills posed a challenge but it was also a potential resource as it provided an opportunity for teams to learn from each other’s experiences. Newly established teams profited from exchanges with well-established teams, but this learning was by no means one-directional as many new teams, unencumbered by history, had fresh and innovative ideas that were well worth replicating elsewhere and could challenge older teams who may have got into a rut and stopped innovating. The same principle of combining expertise and newness was also applied to staff attendance from each team. If the aim of developing teams as a whole was to be achieved, it was crucial that the training included the most senior staff members leading the teams and not just those who were new to EDs.

To promote across team learning, each team was grouped with two other services, geographically accessible to them, for small group work and team development, and each group of teams was assigned a mentor for the duration of the training. Each group of teams was allocated to one of four regional hubs across England in Bristol, London, Manchester, and Peterborough, each of which was led by a member of the steering group.^3^ Twenty-two expert ED clinicians from across England took on the role of mentors to support teams and act as facilitators for the small group work throughout the training.

## Training delivery

During the orientation phase, mentors met individually with each team before the start of the training to understand the current practice of the team, their treatment philosophy, current service configuration, and plans for service developments. The teams were invited to discuss ways in which the team as a whole could create a supportive learning environment as well as discuss the logistics of team participation at external teaching events.

The year-long training programme consisted of 8 days in each hub across England plus a one-day national conference for all four hubs together at the end of the training. Due to the demands of ongoing clinical commitments, not every member of every team attended each training day. Despite this, an average of over 75% of all staff attended on each training day and, except for two teams, experienced, senior members of the team attended regularly.

The training format included a mixture of didactic lectures and experiential and interactive learning in mentor groups. The curriculum covered a wide variety of themes including the epidemiology of EDs, diagnostic criteria, medical risk, assessment, treatment, user participation, and multidisciplinary working (see Additional file 1: table S1 for details).

The training website provided supplementary distance learning which included a series of lectures delivered by leading experts on specific topics, up-to-date clinical and research papers, and video examples of clinical practice. All the lectures that were delivered face-to-face were also recorded and uploaded to the training website. This allowed staff members who were unable to attend all the meetings to keep up with the training.

Throughout the training, mentors provided ongoing support to teams via telephone/video consultations. A mid-point meeting between steering group and mentors was an opportunity to share experiences, discuss team developments, identify improvements that could be made to the programme, and prepare for the second half of the training.

## Conclusions and future developments

The government-funded transformation programme to develop dedicated CEDS-CYP across England is unparalleled in the field of EDs in the UK and arguably among the largest EDs service investments in the world. The National Whole Team Training provided a good foundation to support these service developments. It was always recognized that additional training focussing on specific evidence-based treatments was going to be needed and an important aim of the national training was for each team to identify their particular additional training needs. A great deal of work has also gone into developing regional clinical networks that provide support for the ongoing development of the services and include routine data collection on service use, waiting times, the need for inpatient treatment, etc. although more work is needed to sustain and build on these advances. We will briefly comment on three areas that we see as key to future developments.

### Focused training in NICE concordant evidence-based treatments

There is considerable variability in the proportion of clinical staff in the different CEDS-CYP teams who have been fully trained in the delivery of NICE concordant evidence-based treatments. Some of this training need is met by existing full-time university-based training CYP IAPT courses [64] which provide extended training in both generic family or CBT intervention skills as well as their specific application for EDs. Briefer, more focused courses on the delivery of the manualised treatments recommended by NICE which are suitable for clinicians who already have the more generic therapeutic skills are provided by MCCAED as well as other training providers and are being regularly accessed by CEDS-CYP teams.

The MCCAED trainings [65] follow a similar principle as the National Team Training of focussing on training teams rather than individuals and usually, each training cohort includes several teams from different services. They include trainings in FT-AN, FT-BN as well as trainings in Multi-family Therapy, CBT, treatment of self-harm, management of ARFID, masterclasses, and trainings in the supervision of FT-AN, etc. Since the start of the transformation programme in April 2016, MCCAED has trained over 800 clinicians from more than 70 teams in FT-AN. Due to the COVID-19 pandemic, trainings had to be moved online and we envisage that many of the trainings will continue to be delivered either fully online or using a mixture of online and face-to-face training. Teams also have an opportunity to receive ongoing external team supervision on the implementation of the treatment model.

### Maintaining ongoing learning and development of training networks

There is considerable evidence [66, 67] that the delivery of high-quality evidence-based treatments such as FT-AN requires more than a one-off brief training. Ongoing supervision, supervision of supervision, training and research updates are all part of good clinical practice. In well-established teams, where there are experienced therapists, processes will be evolved to ensure ongoing learning and support for an appropriate balance between adherence to the treatment model and flexibility to meet the specific needs of individual patients and their families [68, 69]. Newer teams may require external input through ongoing supervision/consultation to the team until the evidence-based treatment model is well embedded in the team’s practice. In addition to this, teams will also have different needs for access to basic and/or advanced training in the evidence-based models for new staff members.

To address some of these issues we have adopted a train the trainer model through the development of local training networks. Trainers from these networks initially join MCCAED trainings observers/co-facilitators before running their own training programmes. They also receive supervision of their training from MCCAED staff and attend joint update meetings with training staff from other networks.

### Consolidating service developments

This is the least straightforward area to assess. Most of the newly developing teams have actively embraced the principles for CEDS-CYP services set out in the Access and Waiting Time Standard [1] but there has been a predictable variability in the way different teams have developed. This is to be expected and can be seen as a necessary phase in the development of the new system. Nevertheless, in the first four years since the start of the service transformation programme, good progress was made across the services as a whole, as can be seen by the steadily increasing numbers seen by CEDS-CYP and decreasing waiting times. In the first year of the programme over 5,000 new referrals were seen across England (representing an annual incidence of 9.5/100,000 population) [70]. As the new services became established, there was a steady increase in referrals particularly during the first two years and by year four this had risen to over 8,000 referrals (annual incidence 14.3/100,000). It is worth noting that the growth in referrals only applied to those classified as routine cases but not cases seen as being urgent that remained relatively steady during this period at around 1,000 referrals/year.^4^ At the same time, the numbers meeting the specified waiting times criteria (4 weeks for routine referrals and 1 week for urgent cases) had risen from an average of 65% to over 85% [70]. The overall increase in referrals was in keeping with the findings of the House et al., study [12] that had informed the service model.

The onset of the COVID-19 pandemic, however, had a major impact on further consolidation of the services. Services not only had to make rapid adjustments to working online [71] but also had to deal with a surge in referrals, currently reaching over 12,500/year (incidence 22.3/100,000). Unlike the early growth in referrals, the greatest increase during this surge was in urgent referrals which more than doubled since the onset of the pandemic with the largest surge in referrals coinciding with the lockdowns introduced by government to try to control the spread of COVID-19. Not surprisingly, the increase in referrals has increased waiting times (currently the numbers meeting waiting time criteria has reduced to around 65%) [70]. Nevertheless, the fact that the services have been able to continue to provide treatment for both urgent an routine case at these much larger numbers speaks volumes about the robustness of the service model.

The negative impact of the COVID-19 pandemic on mental health has been well documented with significant increases in anxiety and depression particularly in older and/or more isolated individuals [72]. The impact on EDs appears notably high, with major increase in incidence, particularly of those with more severe presentations and/or rapid deterioration requiring hospitalisation, which has been reported from around the world [73–77]. Recent data has highlighted that in addition to the direct impact of the COVID-19 pandemic on individuals with EDs vulnerability [74, 78], the impact that the pandemic has on family dynamics also appears to play a significant role. While some report a positive effect of an increased connection and greater closeness in the family during lockdown [79], for others, where the impact of the pandemic has resulted in increased tension or conflict in the family, there appears to be an increased risk of vulnerable individuals developing an ED [80].

## Final thoughts

Developing and implementing service level transformation is a complex, iterative process and its long-term success requires ongoing evaluation and audit at multiple levels. Questions of efficacy, effectiveness, and health economic impacts need to be considered alongside broader questions of intervention reach in the real world that are influenced by interactions between specific treatment interventions, the service context and the dynamic evolution of the service system as a whole [81].

The service model that we have described includes an expectation that individual services routinely collect goal-based and other outcomes and feedback measurement as well as system level, across service data collection on service use, waiting times, the need for more intensive treatments etc. In this paper we have described the success of the model in the first years in terms of increasing case identification and meeting planned targets for timely interventions but also the negative effects of the COVID-19 pandemic on these outcomes. Negotiating the impact of the pandemic, is of course only one aspect of an evolving service system. In looking ahead, it is important to take account of new evidence and changes in the service context that require adaptations and modifications of the service model.

One example has been the acknowledgement of the need to include ARFID in the ED care pathway [82], which prompted NHS England to fund a national ARFID pilot. This increased awareness of ARFID, which alongside growing numbers of referrals highlighted the need for additional funding and the need for training in the management and treatment of ARFID. Another example has been the work on reducing the frequency and lengths of inpatient stays for the small number of patients requiring more intensive additional treatment. This required offering alternatives such as brief intensive day care [83], outreach home interventions [84] or intensive outpatient treatment [85] but above all a better integration of the more intensive interventions with the CEDS-CYP model [86].

The COVID-19 pandemic has magnified the pressures that the CEDS-CYP teams have had to address. This has highlighted both some of the weak points in the service model but also, more importantly, the strengths and resilience of the model and has also served as a useful reminder that any service model, however well it has been constructed and implemented, is always a work in progress. Recently NHS England has reconvened an expert working group, which includes the authors of this paper, working with a broader range of experts and stakeholders to refresh the model and the guidance and use the experience gained alongside fresh evidence to consider the whole pathway approach and whether adjustments or enhancements are required to strengthen and improve the service we offer to young people in our community experiencing eating related issues with increasing complexity and acuity.

## Supplementary Information

**Additional file 1: Table S1.** Outline programme of National Whole Team training.

## Data Availability

Not applicable.

## Abbreviations

ED: Eating disorders
RCT: Randomised control trial
NHS: National Health Service
CCG: Clinical Commissioning Group
CAMHS: Child and Adolescent Mental Health Service
MCCAED: Maudsley Centre for Child and Adolescent Eating Disorders
CAEDS: Child and Adolescent Eating Disorders Service
HCHC: House of Commons Health Committee
CEDS-CYP: Community Eating Disorders Service for Children and Young People
CYP IAPT: Children and Young People’s Improving Access to Psychological Therapies
FT-AN: Family therapy for anorexia nervosa
A&WT: Access and Waiting Time Standard

## Footnotes

1 This was for England rather than UK as a whole. The initial funding was for a 5-year period but later was changed to recurrent funding and there have been additional increases since then to £41 million in 2019/20, £52 million in 2020/21 and around £53 million in 2021/22. The increases were to meet rising demand on services, but also to include ARFID (which by then was clearly to be included in ICD-11).

2 At the time the training was set up there were 72 services, but this number has varied as a few services were only set up at a later date and some of the smaller services later merged.

3 Ivan Eisler and Mima Simic from MCCAED and Dasha Nicholls and Cathy Troupp from Great Ormond Street Hospital EDs Service.

4 For definitions of ‘routine’ and ‘urgent’ see Access and Waiting Time Standard [1].
