# Supplementary material for: Implementing service transformation for children and adolescents with eating disorders across England: the theory, politics, and pragmatics of large-scale service reform
Source: J Eat Disord. 2022 Oct 10;10:146. doi: 10.1186/s40337-022-00665-z (PMC9549853; doi:10.1186/s40337-022-00665-z)
Supplement: Supplementary file 1 — Additional file 1: Table S1. Outline programme of National Whole Team training. [file 40337_2022_665_MOESM1_ESM.docx]

Supplementary Table 1- Outline programme of National Whole Team training

| Topic | Session | Delivery format | Speaker/facilitator |
| --- | --- | --- | --- |
| Day 1 – The Nature of Eating Disorders | | | |
| Pre-recorded *online* lecture to be viewed before this training day: Nadia Micali: ‘Epidemiology of eating disorders’ | | | |
| Orientation | | | |
|  | Delivering a national whole team training for child and adolescent eating disorders: Aims and structure of training | *Lecture to whole group* | Hub leader |
|  | Key issues in the current knowledge base of eating disorders plus new NICE guidelines | *Lecture to whole group* | Hub leader |
| Biological and psychological models | | | |
|  | Genetics and familial risk | *Pre-recorded lecture to whole group* | Janet Treasure |
|  | Genetics – Discussion of lecture | *Small ad hoc groups* | Hub leader |
|  | Neuroimaging in eating disorders | *Pre-recorded lecture to whole group* | Savani Bartholdy |
|  | Brains – Discussion of lecture | *Small ad hoc groups and whole group* | Hub leader |
| Epidemiology | | | |
|  | ‘Social and psychological risk factors in eating disorders | *Pre-recorded lecture to whole group* | Dasha Nicholls |
|  | Risk factors – discussion of lecture | *Small ad hoc groups and whole group* | Hub leader |
| Team working | | | |
|  | Shared team philosophy  Past, present and future of team  Hopes, doubts and fears | *Team subgroups*  *Whole teams*  *Team mentor group* | Team mentors |
| Evaluation of Day 1 | | *Individual teams* | Team mentors |

| Topic | Session | Delivery format | Speaker/facilitator |
| --- | --- | --- | --- |
| Day 2 – Physical Risk Assessment and Management | | | |
| Welcome from hub leader, feedback and reflections on day 1 | | | |
| Starvation | | | |
|  | The biology of starvation | *Lecture to whole group* | Simon Chapman |
| Risk assessment | | | |
|  | Risk assessment in eating disorders | *Lecture to whole group* | Lee Hudson |
|  | Risk assessment – Discussion of lecture | *Whole group* | Lee Hudson and Simon Chapman |
|  | What is healthy weight? | *Lecture to whole group* | Simon Chapman |
|  | Bone mineral density in children and young people with anorexia nervosa | *Lecture to whole group* | Lee Hudson |
|  | Q&A session | *Whole group* | Lee Hudson and Simon Chapmn |
| Risk assessment in clinical practice | | | |
|  | Risk assessment | *Live demonstration* | Lee Hudson and Simon Chapman |
|  | Risk factors – discussion of lecture | *Small ad hoc groups* | Hub leader |
| Group work | | | |
|  | Risk assessment case studies | *Team mentor groups exercise* | Team mentors, Lee Hudson, Simon Chapman and hub leader |
| Evaluation of Day 2 | | *Individual teams* | Team mentors |

| Topic | Session | Delivery format | Speaker/facilitator |
| --- | --- | --- | --- |
| Day 3 – Psychological Assessment, Diagnosis and Consent | | | |
| Pre-recorded online lecture to be viewed before this training day:  ‘Compulsory treatment of children and young people with eating disorders - legal options’ by Sophie Pownall (GOSH solicitor) | | | |
| Welcome from hub leader, feedback and reflection on training so far | | | |
| Diagnosis | | | |
|  | Definitions and diagnosis of eating disorder:   - Revisions of classifications - Restrictive eating disorders - BN, ARFID, BED - Are diagnostic criteria relevant | *Whole group lecture* | Rachel Bryant-Waugh |
|  | Discussion of lecture | *Whole group* | Hub leader |
| Consent and capacity | | | |
|  | Legislation, consent and capacity | *Whole group lecture* | Jacinta Tan and Anne Stewart |
|  | Discussion of lecture | *Whole group discussion and exercise* | Jacinta Tan and Anne Stewart |
| Co-morbidity | | | |
|  | Co-occurring disorders and their treatment in an eating disorders context | *Whole group lecture* | Mima Simic |
|  | Discussion of lecture | *Whole group discussion* | Mima Simic |
| Assessment and initial engagement with the family | | | |
|  | Assessment and engagement | *Video and small group exercise in team mentor groups* | Team mentors |
| Evaluation of Day 3 | | *Individual teams* | Team mentors |

| Topic | Session | Delivery format | Speaker/facilitator |
| --- | --- | --- | --- |
| Day 4 – Working as a Specialist Eating Disorders Team | | | |
| Pre-recorded online lecture to be viewed before this training day: ‘The impact of EDs on family life’ by Ivan Eisler | | | |
| Welcome from hub leader, feedback and reflection on training so far | | | |
| Professional, social and cultural context of treatment of eating disorders | | | |
|  | Key role of MDT in engagement, assessment, management and treatment | *Lecture to whole group* | Ivan Eisler |
|  | Social and cultural factors in treatment of ED   - Societal and professional stereotypes and beliefs, identifying ED and access to services - Acculturation, immigration, racism and ED - Inter-cultural work, families, wider systems | *Lecture to whole group* | Liz Dodge |
|  | Discussion and Q&A | *Whole group* | Liz Dodge and Ivan Eisler |
| The role of families in treating eating disorders | | | |
|  | Eating disorders focused family therapy   - The current evidence base for FT-ED - The use of treatment manuals in clinical practice, their strengths and limitations - Key principles of FT-ED - Key principles of multi-family therapy | *Lecture to whole group* | Ivan Eisler |
|  | Discussion of lecture | *Whole group* | Ivan Eisler and hub leader |
| Developing a treatment plan | | | |
|  | Developing a management and treatment plan that will engage family and young person | *Video observation and small group exercise* | Team mentors |
| Team working | | | |
|  | Team development exercise | *Mixed team subgroups* | Team mentors |
| Evaluation of Day 4 | | *Individual teams* | Team mentors |

| Topic | Session | Delivery format | Speaker/facilitator |
| --- | --- | --- | --- |
| Day 5 – What Makes Therapy Work? Plus Nutritional Management | | | |
| Pre-recorded online lectures to be viewed before this training day:  ‘Genetic risk, appetite regulation & obesity’ by Clare Llewellyn and ‘Why do therapies work’ by Peter Fonagy | | | |
| Welcome from hub lead, feedback and reflection on training so far | | | |
| Evidence-based treatment and common therapeutic factors | | | |
|  | IAPT principles as a template for service transformation   - Improving access and engagement - Delivery of evidence-based practice - Improving outcomes accountability - Youth, carer, and community participation - Raising mental awareness and reducing stigmatisation | *Pre-recorded lecture to whole group* | Peter Fonagy |
|  | Evidence base for child and adolescent eating disorders treatments | *Lecture to whole group* | Dasha Nichols |
| Delivering evidence based treatment in practice: | | | |
|  | Choice of and use of manuals, adherence | *Interactive exercise with whole group* | Hub leader and mentors |
| Nutritional management | | | |
|  | Nutritional management, meal planning, re-feeding syndrome | *Lecture to whole group* | Graeme O’Connor |
|  | The role of dietitians in Community Eating Disorder Teams | *Panel discussion with whole group* | Graeme O’Connor, Hub leader, Mentors, two dietitians from local teams |
| Nutritional management: How do dietetics work alongside therapies? | | | |
|  | Applying nutritional knowledge in assessment and treatment within a range of therapies | *Mixed team subgroups exercise* | Graeme O’Connor, Hub leader, Mentors |
| Evaluation of Day 5 | | *Individual teams* | Team mentors |

| Topic | Session | Delivery format | Speaker/facilitator |
| --- | --- | --- | --- |
| Day 6 – The range of treatments and what to do when evidence-based therapy isn’t working | | | |
| Pre-recorded online lecture to be viewed before this training day:  ‘Cognitive Remediation Therapy for Anorexia Nervosa’ by Kate Tchanturia | | | |
| Welcome from hub lead, feedback and reflection on training so far | | | |
| Other NICE recommended treatments | | | |
|  | CBT | *Lecture to whole group* | Beth Watkins/ Lucy Serpell |
|  | Adolescent Focused Therapy | *Lecture to whole group* | Cathy Troupp/Dasha Nicholls |
|  | What to do when evidence-based treatment isn’t working | *Lecture to whole group* | Mima SImic/ Katrina hunt |
|  | Personality traits, self-harm, DBT and RO DBT | *Lecture to whole group* | Mima SImic/ Katrina hunt |
|  | Parent and carer focused interventions | *Lecture to whole group* | Irene Yi/Dasha Nicholls |
|  | The use of groups in ED treatment | *Lecture to whole group* | Cathy Troupp/Dasha Nicholls |
|  | Discussion and Q&A | *Panel discussion with whole group* | Hub leader+ speaker panel |
| Using a range of therapies: | | | |
|  | Developing evidence-based care pathways /decision trees (part I) if optimum team resources were available | *Individual teams in mentor groups* | Hub leader and mentors |
|  | Developing evidence-based care pathways (part II) with current team resources. What are the gaps and how can resources be best used | *Individual teams in mentor groups* | Hub leader and mentors |
|  | Poster presentations to other teams in mentor group | *Mentor groups* | Lead by teams |
| Evaluation of Day 6 | | *Individual teams* | Team mentors |

| Topic | Session | Delivery format | Speaker/facilitator |
| --- | --- | --- | --- |
| Day 7 – User Participation, Shared Decision-Making, and Outcomes | | | |
| Administrative staff from participating teams invited to this training day | | | |
| Pre-recorded online lecture to be viewed before this training day: ‘Shared Decision Making’, Kate Martin | | | |
| Welcome from hub lead, feedback and reflection on training so far | | | |
| Shared decision-making and goal setting | | | |
|  | Shared decision making, goal setting and goal-based outcomes | *Lecture to whole group* | Duncan Law and Hub leader |
|  | Using goals and goal-based outcomes in practice | *Group exercises with role play* | Duncan Law and Hub leader and mentors |
|  | Administrators facilitated workshop | *Small group* | Mentor |
| Young people’s perspectives on services | | | |
|  | •  Short films  •  Guided Q&A  •  User facilitated discussion | *Whole group* | User facilitator; hub leader and mentors |
| Parents perspectives on services | | | |
|  | Parent film and team discussion | *Mentor groups* | Mentors |
| Evaluation of Day 7 | | *Individual teams* | Team mentors |

| Topic | Session | Delivery format | Speaker/facilitator |
| --- | --- | --- | --- |
| Day 8 – Services in Context | | | |
| Regional commissioners invited to this training day | | | |
| Pre-recorded online lecture to be viewed before this training day:  *Anthony Winston: “Transitions to adult services”* | | | |
| Welcome from hub lead, feedback and reflection on training so far | | | |
| Service commissioning | | | |
|  | National policy and update on eating disorders pathway for young children and young people | *Lecture to whole group* | Anne O’Herlihy, NHS England |
|  | Data collection and outcome reporting | *Lecture to whole group* | Anne O’Herlihy, NHS England |
|  | Commissioner panel discussion | *Discussion with whole group* | Hub leader and commissioners |
| Looking after your team | | | |
|  | Mentalizing in systems of help: developing well connected teams and networks | *Lecture to whole group* | Dickon Bevington |
|  | Looking after your team: supervision | *Discussion with whole group* | Mentors |
| Transitions between services | | | |
|  | Pathways into inpatient units | *Lecture to whole group* | Mima Simic/Dasha Nichols |
|  | Transition to adult services: brief summary | *Whole group* | Hub leader |
|  | Final reflections | *Whole group* | Hub leader |
| Team working | | | |
|  | Your team’s journey so far and next steps | *Individual teams in mentor groups* | Team mentors |
| Evaluation of Day 8 | | *Individual teams* | Team mentors |
